# Health Resorts

**Published:** 1902-08-23

**Authors:** 


### HEALTH RESORTS.

### TUNBRIDGE WELLS.

Although by its name Tunbridge Wells declares its mission as a watering place, it is to be feared that but a very small proportion of those who live there, or who visit the place in search of health, or of the crowd of excursionists who disport themselves on its common of an afternoon, ever take the trouble even to taste the waters which first gave it fame. Yet they are good waters, and do in fact provide what it is so difficult to give in the form of a mere physic, namely, a solution of carbonate of iron. According to Sir Hermann Weber, M.D., the water of Tunbridge Wells belongs to the pure chalybeate class; and Dr. Stevenson in an analysis which he made in the year 1891 says that it contains about ·06 per mille of carbonate of iron. Comparing the water with that of certain well-known foreign spas, it is to be noted that the iron it contains occurs in the same form as in some of the springs at Spa, Schwalbach, and St. Moritz. These several places may be arranged in the following order in regard to the strength of their waters, namely—Spa, Schwalbach, Tunbridge Wells, St. Moritz. Unfortunately for Tunbridge Wells its water does not contain that free supply of carbonic acid gas to which so much of the popularity of these other waters is due. The Tunbridge Wells waters are of especial service in cases of anæmia and debility, and being placed in the neighbourhood of a moderately hilly district, in which there are many temptations to take walking and riding exercise, they are likely to be useful by those who will exercise the energy to go down to the Pantiles to drink them in proper form; although in cases of pronounced chlorosis, in which some authorities recommend that very large doses of iron should be taken, it will probably be found desirable to supplement the waters with some pharmaceutical preparation of iron. To obtain the full benefit of the waters they should be taken, as they are at Spa, early in the morning and on an empty stomach, and plenty of time should be spent drinking the prescribed dose. They may also be taken in the middle of the forenoon and again in the afternoon.

Putting the waters on one side, there is a great deal at Tunbridge Wells to make it not only an attractive but a useful health resort. It is a considerable town, the outlying parts of which extend over a very large area, and in the way of churches, amusements, lectures, libraries, etc., it has many attractions not always to be met with in even larger centres of population. At the same time its peculiar position, with its large commons and its hilly slopes, gives it the pure air of the country. Indeed we fancy that the verdict of most of its visitors will be that its attractions lie far more in its air than in its water. Some parts of the town stand high (upwards of 400 feet above the sea level), while all around are hills of varying altitude. It is an open, breezy, and very sunny place, well suited for many kinds of debility, and admirably adapted as a residence for elderly people—especially for those who can afford carriages and horses and comfortable homes, for the winters are cold enough. The town has an enterprising corporation, which supplies electric light, a telephone service, water supply, sewage works, isolation hospital, and other means to health, and its death-rate is phenomenally low.

### ILFRACOMBE.

Ilfracombe is a place to which not only do people go for health, but to which healthy people go, and this is a great virtue, for, especially in the case of neurotic and "run down" individuals, a too invalid society is by no means conducive to recovery. As a health resort Ilfracombe appears to fulfil a double function. On the one hand, it is a place which possesses a curiously equable climate, warm in winter yet always more or less "bracing," while in summer the heat is gently tempered by the Atlantic breezes which blow up from the west. Indeed its position on the Devonshire peninsula, and itself again jutting out into the sea, gives its climate a distinctly oceanic character. Thus, for that large number of cases in which extremes are ill borne, and which, nevertheless, call for air that moves, Ilfracombe is peculiarly well suited. On the other hand, in summer Ilfracombe offers many attractions to those who, while loving the sea, do not wish to have a month of sea alone. For at Ilfracombe there are hills and cliffs and beautiful scenery, with a charming country behind, and many places of interest within visiting distance. Thus to the convalescent who does not wish to be exposed to the risks of a variable climate, yet to whom a mere seaside parade spells boredom, Ilfracombe is a delightful refuge. As one of its great attractions from a purely health point of view, we must refer to the excellent opportunities which it affords of taking sea trips in well-appointed steamers—not mere runs out to sea and back again, such as the melancholy hour on the briny obtainable at some sea-side towns, but excursions to other places, such as to Tenby, Cardiff, Minehead, Clovelly, etc., places where there is something to see. To the absolute invalid these things may be of small interest, but to the neurotic and the convalescent, to whom health seeking is too often at least but a weary business, these little excursions on the sea not only afford a pleasant break but in fine weather are actually health giving.

Turning now to certain details concerning which the careful seeker after health always desires information: According to the Report of the Medical Officer for the year ending December 31st, 1901, the soil is mostly light and thin. The rocks are shales over sandstone and grit, but there are a few pockets of mountain limestone and some deposit is of a poor clay. The southern boundary of the Urban Sanitary District is a range of high ground (600 to 800 feet) forming the water parting and running east and west. The northern boundary is formed by the Bristol Channel. The marine slope terminates abruptly in a line of weatherworn cliffs, which have been rendered rugged and picturesque by continued denudations, and is cut up by three valleys trending north and south. The average winter temperature is 44 9; and for the three months of February, March, and April 44 6. The average summer temperature is 57° Fahr. The daily range of temperature is 8·4° Fahr. The water supply is constant and of good quality. At present it is collected from the hills in the neigbourhood of Morthoe and is filtered, but the engineers are actively engaged in providing an additional supply direct from the forest of Exmoor. The sewage is disposed of by being discharged into the tideway where there is a good current, but with the growth of the town the flushing of sewers has become somewhat of a difficulty in some parts, and one of the advantages which it is hoped will be gained by an additional water supply will be the greater facilities for the complete flushing of the sewers which will be thereby afforded. The sewers and their outfalls, etc., have been maintained in good condition, and it has been resolved to extend the outfall and to relay almost all the older sewers. In the meantime the house connections are carefully inspected and tested. When these improvements and the new water supply have been completed there is no doubt that the drainage of the town will be placed on a satisfactory footing. The town possesses a proper sanitary machinery, two isolation hospitals, a Thresh's steam disinfector, and the arrangements for scavenging, etc., seem to be carefully and effectually organised. Judged by its death-rate Ilfracombe appears to be a healthy place, the gross death-rate for the past year having been 13·8, while after deducting the deaths occurring amongst strangers the true nett death-rate works out at only 12·5. Moreover, there were no deaths from any of the scheduled infectious diseases, and only four from the other zymotics, and those were from diarrhœa. There are no factories in Ilfracombe, and no offensive trades, and there are no common lodging houses within the district. In the year 1899 there was a certain amount of diphtheria in the place; but, as the report states, there were no notifications of that disease last year after the month of July, and there were no deaths from it at all last year, so the outbreak may be taken to have passed away. Evidently Ilfracombe under the intelligent guidance of its medical officer is putting its house in order, and no one can doubt that with its extraordinary natural advantages it is likely to maintain its position as a most popular and attractive health resort.

